# Supplementary material for: Amelioration of Scopolamine-Induced Cognitive Dysfunction in Experimental Mice Using the Medicinal Plant Salvia moorcroftiana
Source: Brain Sci. 2022 Jul 7;12(7):894. doi: 10.3390/brainsci12070894 (PMC9320495; doi:10.3390/brainsci12070894)
Supplement: Supplementary file 1 [file brainsci-12-00894-s001.zip › brainsci-1708808-supplementary.pdf]

Supplementary file

Amelioration of scopolamine induced cognitive dysfunction in experimental mice using the medicinal plant, *Salvia moorcroftiana*

Fazal Wahid[1], Tour Jan[1, *], Fakhria A. Al-Joufi[2], Syed Wadood Ali Shah[3], Mohammad Nisar[1], Muhammad Zahoor[4,*]

Table S1. Cholinesterase inhibitory potentials *S. moorcroftiana* and fractions.

Test Sample	Cholinesterase inhibition IC_{50} (µg/mL)	
	AChE	BChE
SlMo-Crd	1120.21	1054.71
SlMo-Hex	1739.82	1705.20
SlMo-Chl	626.92	547.12
SlMo-Et	759.30	711.77
SlMo-Bt	1398.71	1109.61
SlMo-Aq	1186.26	1081.76
Donepezil	4.96	3.80

All values are presented as mean±SEM, *n*=3, SlMo-Crd: crude extract, SlMo-Chl: chloroform fraction; SlMo-Et: ethyl acetate SlMo-Bt: butanol and SlMo-Aq: aqueous fraction of *S. moorcroftiana*.

Table S2. Effect of SlMo-Crd and fractions on AChE and ACh level in brain.

Sample Test		AChE (µmoles of substrate hydrolysed / min / g tissue)		ACh (mmol/min/mg protein)	
		HC	FC	HC	FC
Control		13.44±1.21	11.09±1.13	17.02±1.29	14.22±1.23
Amnesic control (Scopolamine)		30.87±1.32§§§	25.61±1.29‡‡‡	6.21±1.14§§§	5.78±1.07‡‡‡
SlMo-Crd	100mg	17.44±1.56*	15.67±1.41*	12.78±1.40*	12.34±1.28**
	200mg	17.32±1.43**	15.20±1.35**	13.29±1.35**	12.88±1.30**
SlMo-Chl	75mg	16.36±1.45***	13.37±1.49**	14.45±1.52***	13.88±1.25***
	150mg	16.01±1.61***	13.01±1.38***	14.81±1.38***	14.10±1.34***
SlMo-Et	75mg	16.67±1.48**	14.49±1.21**	13.87±1.27**	12.93±1.41**
	150mg	16.22±1.39**	14.11±1.50***	14.69±1.56***	13.11±1.29***
Donepezil	2mg	12.96±1.31***	11.87±1.22***	16.14±1.51***	14.34±1.40***

Mean±SEM (n=6). Oneway ANOVA after which Dunnett's post hoc multiple comparison test to estimate the values of P. §§§ P<0 001, ‡‡‡P<0 001 comparison of scopolamine treated (amnesic) group vs. normal control, *P<0.05, **P<0.01 and ***P<0.001 as comparison of scopolamine treated (amnesic) group vs. Donepezil, crude extract and fractions-treated groups, by using one way ANOVA followed by Dunnet comparison. Crude extract (SlMo-Crd), chloroform (SlMo-Chl) and ethylacetae (SlMo-Et) fraction.

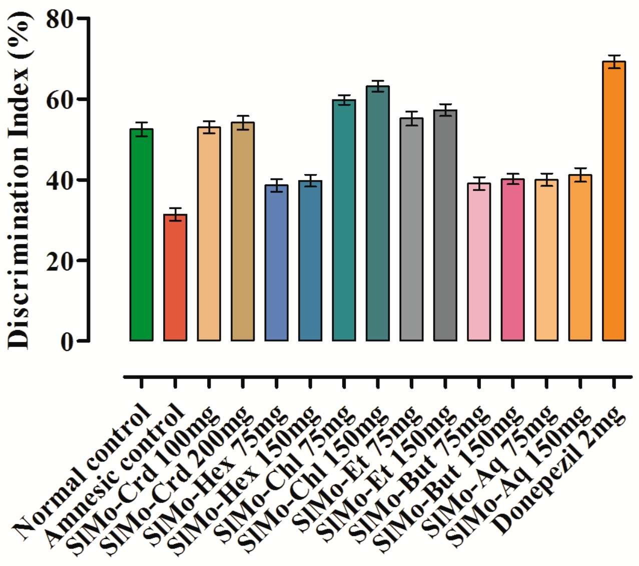

Figure S1. Discrimination index of SlMo-Crd and fractions using NORT.
